# A Synopsis of Current Theories on Drug-Induced Nephrotoxicity

**DOI:** 10.3390/life13020325

**Published:** 2023-01-24

**Authors:** Lukasz Dobrek

**Affiliations:** Department of Clinical Pharmacology, Wroclaw Medical University, Wroclaw, Poland; lukasz.dobrek@umw.edu.pl or xlukaszx@onet.eu

**Keywords:** kidney, drugs, nephrotoxicity, adverse drug reactions

## Abstract

The overriding goal of the treatment of patients is its effectiveness and safety. However, all medications currently being used also exert some adverse pharmaceutical reactions, which may be regarded as an unintended but inevitable cost of pharmacotherapy. The kidney, as the main organ that eliminates xenobiotics, is an organ especially predisposed and vulnerable to the toxic effects of drugs and their metabolites during their excretion from the body. Moreover, some drugs (e.g., aminoglycosides, cyclosporin A, cisplatin, amphotericin B, and others) have a “preferential” nephrotoxicity potential, and their use is associated with an increased risk of kidney damage. Drug nephrotoxicity is, therefore, both a significant problem and a complication of pharmacotherapy. It should be noted that, currently, there is no generally recognized definition of drug-induced nephrotoxicity and no clear criteria for its diagnosis. This review briefly describes the epidemiology and diagnosis of drug-induced nephrotoxicity and characterizes its pathomechanisms, including immunological and inflammatory disturbances, altered kidney blood flow, tubulointerstitial injury, increased lithogenesis–crystal nephropathy, rhabdomyolysis, and thrombotic microangiopathy. The study also lists the basic drugs with nephrotoxicity potential and provides a short overview of the preventive methods for reducing the risk of drug-related kidney damage developing.

## 1. Introduction

The kidneys are small organs (approximately 165 ± 40 [g] in males and 122 ± 29 [g] in females) located retroperitoneally on the posterior abdominal wall and found between the transverse processes of T12 and L3. Human kidneys account for only about 0.2–0.5% of total body weight, but they receive as much as 20% of the cardiac output. Despite their small size, kidneys are essential for life due to the high renal blood flow value and the role of the kidneys as the main point of elimination of xenobiotics, potentially nephrotoxic substances that are delivered to the organ in relatively high concentrations. Thus, it is reasonable to assume that the kidneys and urinary tract are privileged targets of the noxious actions of drugs and other xenobiotics [1].

Kidneys carry out a lot of integrated functions aimed at maintaining the homeostasis of the body, including: the control of the composition and volume of body fluids; electrolytes and acid-base balance; the long-term regulation of blood pressure in a sodium-dependent manner; and the synthesis and secretion of several hormones (e.g., renin, eicosanoids, erythropoietin, and 1,25-dihydroxyvitamin D3) [2,3]. Moreover, the kidneys and the urinary system are key in eliminating xenobiotics, including drugs. The elimination process of these compounds consists mainly of the excretion of parent substances and their metabolites in the urine (by glomerular filtration and tubular secretion). Furthermore, the elimination activity of the kidneys results from their ability to metabolize drugs and other xenobiotics, although the function is limited compared to the biotransformation process taking place in the liver. Nevertheless, the kidneys also express a metabolic cytochrome capacity. A growing body of evidence indicates that CYP 2B6 and 3A5 are expressed in human kidneys, mostly in the proximal tubules, whereas CYP 1A1, 1A2, 1B1, 2A6, 2C19, 2D6, and 2E1 are not found in human kidneys in significant amounts, whilst the presence of CYP 2C8, 2C9, and 3A4 in human kidneys is still being researched [4]. However, other studies have confirmed the activity of CYP1A1, CYP2C8, CYP2C11, CYP2E1, and CYP3A5 in human kidneys. In addition, the organ contains isoenzymes of the CYP2J subclass, physiologically absent in the liver, and flavin monooxygenases which catalyze the biotransformation of drugs with functional groups containing nitrogen, sulfur, and phosphorus atoms. The kidneys also contain other enzymes that metabolize drugs, such as monoamine oxidases (MAO-A and MAO-B), alcohol and aldehyde dehydrogenases, and prostaglandin H complex synthases [4,5,6]. To summarize, the role of the kidneys as the first oxidative-reduction phase of biotransformation is well documented, although there is still debate about the qualitative and quantitative assessment of the presence of individual cytochromes in kidney tissues. Phase II reactions of drug metabolism (the coupling of metabolites with specific carriers (e.g., glucuronide) that facilitate their excretion into the urine) also occur in the kidneys. UDP-glucuronosyltransferases (UGTs), glutathione S-transferase, and N-acetyltransferase have also been found in the kidneys. The most abundantly expressed renal UGTs are 1A9 and 2B7, which are involved in the glucuronidation of drugs, arachidonic acid, prostaglandins, leukotrienes, and P450-derived arachidonic acid metabolites [4,5,6,7].

Drugs and their metabolites are eliminated from the body in the urine by both glomerular filtration and tubular secretion. The ultrafiltrate formed as a result of glomerular filtration is modified by the tubular transport taking place mostly in the proximal tubules. In the context of nephrotoxicity, it is important that the tubular cell uptake of potentially nephrotoxic compounds occurs via the apical (via endocytosis/pinocytosis and other passive or active transport pathways) or basolateral (via the peritubular capillaries) membranes of the proximal tubules. In the process of tubular uptake, the family of active transporters, being organic anion transporters (OAT) for negatively charged drugs and organic cation transporters (OCT) for positively charged drugs, as well as sodium dicarboxylate transporters or other active transport pumps, are involved [8]. Overall, due to the metabolic role of the kidneys in drug biotransformation, renal clearance is the dominant component of total clearance for most drugs. The kidney itself is a privileged target of their noxious action and numerous drugs may produce renal-adverse reactions. The general rationale for the thesis is an assumption that numerous kidney diseases significantly influence the pharmacokinetic profile of drugs, especially the stages of excretion and, in part, the metabolism. On the other hand, however, drugs and their metabolites excreted by the kidneys may themselves cause functional and/or structural renal dysfunction due to their nephrotoxicity potential. The proximal tubules are the most sensitive to the nephrotoxic effects of xenobiotics and drugs, as they are characterized by the highest metabolic activity, are high on the osmotic gradient, as mentioned above, and due to the presence of tubular transport systems involved in the excretion and resorption of compounds from primary urine ultrafiltrate.

## 2. Epidemiology and Diagnosis of Drug-Induced Nephrotoxicity

Drug-induced nephrotoxicity, also less frequently named drug-induced kidney disease (DIKD), is a common clinical problem. It is estimated to affect as many as 14–26% of adult and 16% of pediatric patients [9]. However, the incidence of drug-related nephrotoxicity maybe even as high as 66%. Among older adults, the incidence is higher because these patients are characterized by multiple diseases and polypharmacy and are, thus, subjected to multiple diagnostic and therapeutic procedures that have the potential to damage the kidneys (e.g., the use of contrast agents in diagnostic imaging). Drug-related kidney dysfunction is often reversible and resolves upon the discontinuation of the offending drug; however, it may also produce irreversible structural damage to kidney tissue. Nephrotoxicity manifests itself over a wide spectrum, reflecting damage to different nephron segments, including glomerular and tubular targets. Ultimately, drug-induced nephrotoxicity is one of the etiopathogenetic factors of either acute kidney injury or chronic kidney disease (CKD) [10].

Although there is no standard definition, DIKD is defined as any damage to the kidneys caused directly or indirectly by drugs, which leads to kidney dysfunction. DIKD is usually classified according to the predominant pathomechanism responsible for kidney damage development or based on the time of the course, with the latter division including both acute and chronic DIKD forms. Acute DIKD is recognized as a 0.5 mg/dL or 50% rise in serum creatinine over a 24–72 h time frame after a minimum of 24–48 h of drug exposure [9,11]. Based on the time course and duration of kidney dysfunctions, Mehta et al. proposed clinically classifying drug-related nephrotoxicity into acute (1–7 days), subacute (8–90 days), and chronic (persistence of > 90 days; the development of chronic kidney disease) [12]. These categories were adapted from conceptual models proposed by the Kidney Disease Improving Global Outcomes (KDIGO) for Acute Kidney Disease (AKI). Injury to the kidney beyond 7 days but less than 90 days reflects sub-acute injury similar conceptually to acute kidney disease as proposed in KDIGO guidelines [13]. However, because the drug-related kidney disturbances may appear delayed, and because the underlying kidney and/or systemic disturbances are being treated that cover symptoms of increasing kidney damage, the recognition of drug-induced nephrotoxicity is clinically difficult since other risk factors could be significant to the attribution of global nephrotoxicity risk.

**Figure 1:**
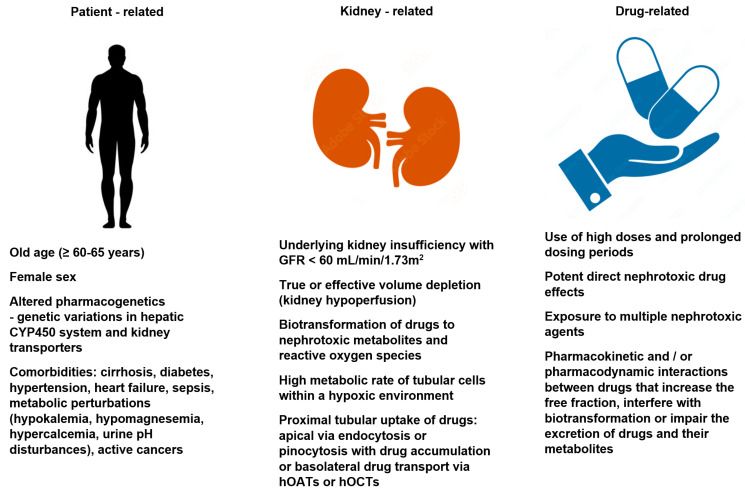
Risk factors for drug-induced kidney disease development [8,9,10]. hOAT—human organic anion transporters; hOCTs—human organic cation transporters.

Considering the problem of the difficult clinical description of drug nephrotoxicity and a lack of an unequivocal consensus on its diagnosis, a novel framework of drug-related nephrotoxicity diagnostic and therapeutic procedures has been proposed by Awdishu and Mehta, which focuses on risk assessment, early recognition, targeted response, timely renal support and rehabilitation, and research into the epidemiology and pathophysiology—otherwise known as the rules of the “6R’s of drug-induced nephrotoxicity” [11]. They are briefly described below:

Risk: Risk factors for drug-induced nephrotoxicity include patient, kidney, and drug-related factors. They are listed in Figure 1. The knowledge of these risk factors and taking preemptive measures (mentioned later in the paper), coupled with vigilance and the early recognition of kidney dysfunction, allows for the effective prevention of the development of drug-induced nephrotoxicity. Patient-related risk factors may be non-modifiable (e.g., age, sex, genetic variation) or at least partially modifiable (e.g., chronic comorbidities or metabolic perturbations). The role of genetic predisposition in the pathomechanism of drug-induced nephrotoxicity is still under debate. Some premises suggest that metabolic pathways and drug transporters vary between patient populations due to the possible polymorphisms of the genes encoding the pharmacokinetic processes. Both the elderly and the female sex are associated with a decreased lean body mass, reduced total body water, and a decreased serum albumin concentration (hypoalbuminemia), and these factors are associated with an increased risk of the elevation of free drug fraction. Moreover, the elderly have been shown to have higher circulating angiotensin II and endothelin levels that contributes to the vasoconstriction of the nephron efferent arterioles, increasing glomerular filtration and the amount of drugs and metabolites reaching the tubules [8]. One of the most important risk factors for drug-related nephrotoxicity is the significant innate kidney toxicity of the offending agent. Some drugs (e.g., aminoglycosides, amphotericin B, polymyxins, cisplatin, cyclosporine, and contrast dye) have a high potential for nephrotoxicity, and so, impaired renal function during treatment may develop as a result of their exposure to the kidneys, even in patients with minimal or no additional risk. Drug-induced kidney disease can also develop as an unwanted and unpredictable adverse drug reaction type B, i.e., an immune response (“bizzare”) that is dose-independent. The immune-related mechanisms include Gell–Coombs hypersensitivity reactions, pseuroallergic reactions, and idiosyncratic reactions. However, the vast majority of drug nephrotoxicity is a dose-dependent adverse drug reaction type A and/or associated with the prolonged duration of treatment. Dose-dependent reactions are predictable and based on the pharmacological properties of the drugs [8,9,10].

Recognition: In order to improve the diagnosis of kidney drug-related dysfunction, it was assumed that the phenomenon may take the form of one of four phenotypes based on clinical presentation, these being acute kidney injury, glomerular disturbances, tubular disturbances, and nephrolithiasis. Primary and secondary clinical criteria should be revealed to support the specific phenotype recognition, and in the case of acute kidney injury, acute kidney disease, and chronic kidney disease, an appropriate timeframe based on the KDIGO findings characteristic for these diseases should also be demonstrated [11,12]. The brief characteristics of the above-mentioned phenotypes are given in Table 1. The criteria for acute kidney injury adopted in the proposed drug-induced nephrotoxicity phenotypes are consistent with the general guidelines based on the current KDIGO guidelines [13].

In each of the particular phenotypes listed in Table 1, at least one primary criterion should meet the Bradford-Hill causal criteria. These criteria were initially introduced in 1965 to determine if the observed associations are of a causal nature. The Bradford-Hill criteria described a number of general features that characterize a causal relationship between an exposure and an outcome, and they quickly became a fundamental tenet in epidemiology [14,15]. Firstly, in terms of the diagnosis of drug-related nephrotoxicity based on the causal Bradford-Hill criteria, it must be highlighted that the drug exposure must precede symptom development by at least 24 h, and there must be an explanation for a drug’s causal role in kidney injury based on the drug’s known pharmacodynamic properties, metabolism, and immunogenicity. In addition, complete data on comorbidities, surgical procedures, blood pressure, urine output, and other factors acting on the patient during the period of drug exposure should be collected to clearly determine the additional risk of nephrotoxicity. Finally, taking into account all the collected data, the relationship between drug use and the development of a given kidney injury phenotype may be considered [11].

Response: The response to the treatment depends on the phenotype, the severity of the injury, and the underlying disease indicative of the particular drug. Therefore, during the treatment of nephrotoxicity, concurrent risk factors for kidney injury must be taken into account, such as hypertension, hyperglycemia, and anemia; in order to minimalize the possible drug interactions that could worsen kidney damage [11].

Renal support: Implementing kidney support (kidney replacement therapy; dialysis or hemodialysis) during the treatment of kidney dysfunction enables the removal of the offending drug from the blood, minimalizing the resulting damage and supporting the kidneys in achieving their recovery. However, the decision to subject a patient to dialysis/hemodialysis in the course of drug-induced kidney dysfunction is complex, and the procedure is usually only implemented in patients with severe kidney damage and in the case of nephrotoxic drugs, characterized by a sufficiently high free-fraction that can be removed by dialysis [11].

Renal rehabilitation: Rehabilitation and the clinical monitoring of patients presenting drug-induced nephrotoxicity symptoms involve the repeated evaluation of the kidney functions in order to assess the potential reversibility and to avoid re-exposure to the offending drug. Furthermore, the aim is to report all of the adverse effects that may have occurred during the administration of potentially nephrotoxic agents. A valuable diagnostic tool facilitating the individualization of treatment and contributing to improving the safety of therapy is therapeutic drug monitoring (TDM) in the blood. The procedure should be employed, if available, as it may prevent the further development of nephrotoxicity [11]. As already mentioned above, nephrotoxicity is the main complication during treatment with many antibiotics, particularly aminoglycosides, vancomycin, or amphotericin B. Alqahtani et al. studied TDM for antibiotics and the association between the non-adherence to TDM guidelines and the development of nephrotoxicity. They demonstrated that adherence to TDM guidelines improves clinical practice and contributes to the reduction of the cost associated with the development of nephrotoxicity. Thus, TDM use may ultimately reduce the cost of treatment of kidney dysfunctions [16].

Research: The last “R” in the approach proposed by Awdishu and Mehta [11] is research. It is emphasized that further research on epidemiology and diagnosis is necessary since there is still no broad consensus regarding the understanding of drug-induced nephrotoxicity and generally accepted guidelines for this illness. New research is needed that focuses on new risk factors for nephrotoxicity, especially as they differ from drug to drug. The still unsolved problem of the cause-and-effect relationship between the drug and an episode of renal dysfunction is also a big challenge. Moreover, there is a lack of experimental, translational studies explaining in detail the pathogenesis of drug-related nephrotoxicity. Finally, the post-authorization of pharmacovigilance is of key importance in the context of the possible disclosure of nephrotoxicity in currently marketed drugs, which has not been proven in their clinical trials [11].

In addition to the above-mentioned classification, based on the 4 main drug-induced nephrotoxicity phenotypes proposed by Awdishu and Mehta [11], there is also Bartoli’s proposal, distinguishing 10 types of drug-related kidney disturbances [17]. This classification includes (1) immunologic reactions caused by drugs involving the kidney (both by direct kidney and systemic immunologic reactions or by hapten-mediated mechanisms), (2) direct toxic effects of drugs on tubular epithelial cells, (3) gadolinium-related renal failure, (4) drug-induced glomerular disease, (5) selective toxic effects of drugs on the kidney, (6) drug-induced hemodynamic alternation of the kidney, (7) crystalline nephropathy, (8) analgesic nephropathy, (9) herb medication-related kidney damage, and (10) adverse drug reactions with different mechanisms manifested by miscellaneous entities involving the kidneys. Some of the drug-induced kidney injuries distinguished by Bartoli [17] are consistent with those reported by Awdishu and Mehta [11], e.g., crystalline nephropathy or the direct toxic effects of drugs on tubular epithelial cells (Bartoli) that correspond to tubular dysfunction (Awdishu and Mehta). The wider classification of Bartoli, taking into account the greater number of types of drug-induced side-effects, results from the identification of certain specific clinical circumstances (e.g., kidney damage after certain herbal drugs or gadolinium-induced renal failure resulting from systemic fibrosis involving the kidneys), which are conditioned by several complementary main mechanisms, also highlighted by Awdishu and Mehta [11].

## 3. Mechanisms of Drug Nephrotoxicity and Examples of Offending Drugs

In general, several mechanisms underlying the development of drug-related nephrotoxicity can be distinguished: inflammatory and immune-related mechanisms leading to acute or chronic glomerulonephritis or interstitial nephritis; altered intraglomerular hemodynamics, triggering acute kidney injury; tubular cell toxicity with the risk of acute tubular necrosis; crystal nephropathy; and rhabdomyolysis or thrombotic microangiopathy.

Inflammatory and immune mechanisms: Drugs can cause inflammatory and immune reactions in all segments of the nephrons, such as in the glomerulus, tubular cells, and the interstitium. The immunocomplex and hapten-mediated mechanisms may be recognized, and drugs can induce the production of antibodies against them and form immune complexes. These complexes can deposit in the glomeruli, contributing to glomerular damage (glomerulonephritis), but also “leak” into the interstitial microcirculation, binding to the basal membrane of the renal tubules, initiating interstitial inflammation. The most reported causative agents for glomerulonephritis are gold compounds, hydralazine, lithium, propylthiouracil, and non-steroidal anti-inflammatory drugs (NSAIDs) [10]. Some drugs (e.g., alpha-methyl dopa, penicillamine, levamisole, or procainamide) may trigger a lupus-like syndrome. The drug-containing immune complexes may also cause a systemic immunologic response leading to microangiopathic vasculitis with kidney involvement. Moreover, drugs can be low-molecular-weight haptens that covalently bind to larger molecules, such as proteins, to form antigenically active substances. These proteins can be in circulation or could be tissue-specific and accumulate in the kidneys. The process can be two-step, i.e., the drug can act as a “prohapten”, requiring endogenous biotransformation into a full hapten. The antibody–hapten complex can also bind to the basal membrane of the renal tubules or interstitial matrix and initiate acute interstitial nephritis (AIN). Usually, the entity develops in a dose-independent manner and does not always have to manifest with the classic symptoms of hypersensitivity (fever, rash, eosinophilia). There are numerous drugs implicated in the AIN pathogenesis: NSAIDs, proton pump inhibitors, beta-lactams (particularly methicillin, quinolones, rifampin, vancomycin, sulphonamides), antivirals (particularly acyclovir and indinavir, thiazides), and loop diuretics [10,17,18,19,20,21]. Antimicrobial agents and NSAIDs are the most frequently offending agents in drug-induced AIN. In antibiotic-induced AIN, hypersensitivity manifestations (skin rash, eosinophilia, fever) are often demonstrated, but oligosymptomatic presentations that are increasingly recognized in the elderly, and are treated with NSAIDs or proton pump inhibitors, are more common nowadays [22]. The other causative factors of AIN involve kidney infections or systemic diseases affecting kidneys (e.g., sarcoidosis, Sjögren syndrome, and systemic lupus erythematosus). AIN represents a possible causative agent of AKI, particularly in hospitalized patients. On the other hand, transient AIN may subside after drug discontinuation or become a chronic entity, even progressing to the development of interstitial fibrosis with subsequent CKD demonstration due to the characteristic interstitial infiltrates, mostly composed of lymphocytes, macrophages, eosinophils, and plasma cells. A significant proportion of AIN cases have an oligosymptomatic presentation, although the presence of specific extrarenal symptoms such as fever, skin rash, and peripheral eosinophilia, with oliguria occurring in up to 50% of patients, may facilitate the diagnosis. Urinalysis in infection-related AIN usually demonstrates proteinuria, hematuria, and pyuria, with white and red blood cell casts [23,24,25]. Inflammation in the kidneys can also become chronic and is diagnosed based on biopsy; interstitial inflammation is present against a background of fibrosis and sclerosis of the glomeruli. Interstitial changes consist of infiltration with mononuclear cells in a matrix that is expanded with increased amounts of collagen, proteoglycans, and fluid. Clinically, chronic interstitial nephritis is manifested mostly by a urinary concentration insufficiency (secondary to dysfunction of the kidney concentrating mechanism located in the medulla) and is usually characterized by low-grade proteinuria and a slow progressive decline in the glomerular filtration rate. This entity contributes to CKD development [26,27]. Drug-related chronic interstitial nephritis is even more difficult to diagnose because it is insidious in its onset, and its course for years has been lacking in both obvious clinical symptoms and urine changes, thus, it has been undetected for a long time. Such a reaction has been reported especially for Chinese herbs containing aristocholic acid and NSAIDs.

The so-called Chinese herb nephropathy was reported for the first time in young Belgian women who were using Chinese herbal slimming remedies. Since then, patients with aristolochic acid nephropathy have been reported worldwide. In vivo and in vitro experiments have revealed some mechanisms of aristolochic acid nephropathy, such as endoplasmic reticulum stress and injury, increasing oxidative stress injury, initiation and sustaining an immune-mediated inflammatory mechanism, and kidney tubular epithelial cell transdifferentiation. Microscopically, the typical findings involve extensive interstitial fibrosis with atrophy and loss of tubules located in the superficial cortex and progressing toward the inner cortex. The interstitium is usually significantly hypocellular, and glomerular lesions mainly include ischemic, microcystic, obsolescent glomeruli, occasional thrombotic microangiopathy-like lesions, and/or focal segmental sclerosis-like lesions. Moreover, apart from nephrotoxicity, aristocholic acid has been proven to be implicated in the genesis of urothelial carcinoma occurring in the renal pelvis and upper ureter [28,29].

The chronic, long-term administration of non-opioid analgesics (NSAID, aspirin, acetaminophen–paracetamol) is linked with an increased risk of chronic tubulointerstitial nephritis development. The chronic ingestion of these drugs in high doses (more than 1 g daily for more than two years) is believed to result in chronic interstitial inflammatory changes that disrupt the vascular system causing ischemia and leading to scarring and fibrosis. The outcome is papillary necrosis and CKD development [10,17,18,19,20,21]. The development of analgesic nephropathy is even more likely because most drugs from this group are available as OTC preparations, so the availability of these agents is basically unlimited. Thus, the true prevalence of AN is difficult to estimate and also because the clinical presentation can vary from asymptomatic hematuria, sterile pyuria, or proteinuria, to symptomatic anemia. The incidence of analgesic nephropathy varies in many countries, which is indirectly related to the availability of NSAID drugs. A study of the Swedish population (people over 65 years of age) showed that 6% had used NSAIDs chronically. Among them, 78% used these drugs as OTC. Almost half of them had a GFR value of fewer than 60 mL/min/1.73 m2 [30]. In an extensive epidemiological study conducted in Australia and New Zealand, based on data from 1971 to 2005, the incidence of analgesic nephropathy was estimated at 10.2% [31]. This was consistent with other findings estimating the global incidence of analgesic nephropathy as being 10% in the late 1990s. Reports also indicate that this disorder is more common in female patients and is associated with the more frequent use of NSAIDs in women compared to men [32]. The most important and accepted issue in the pathophysiological description of analgesic nephropathy is the decline of the vasodilatory effect of prostaglandins, leading to hypoperfusion-related medullary ischemia, which is generally accompanied by papillary damage in the form of necrosis [33]. However, there are also other complementary mechanisms for the damaging effects of NSAIDs on the kidneys. Under physiological conditions, prostaglandins, through the stimulation of tubular EP1 receptors, inhibit the transport of sodium and chloride in the ascending loop of Henle and in the collecting ducts, leading to natriuresis. In addition, prostaglandins exert an antagonistic action on the antidiuretic hormone (ADH) receptors and contribute to an increase in diuresis; therefore, NSAID abuse may cause higher sodium and water retention by inhibiting PGE2 production, leading to fluid overload and predisposing to the development of capacitive arterial hypertension [33,34]. NSAIDs have also been shown to be associated with the intensification of oxidative stress and the production of reactive oxygen species (ROS), and this pathomechanism also contributes to kidney damage [35]. It seems to be of importance, especially for paracetamol toxicity. When paracetamol is abused and used with other non-opioid analgesics, especially aspirin, the preventive detoxification mechanism of ROS is depleted, and ROS can cause peroxidative damage to kidney tissues [33].

Altered intraglomerular hemodynamics and acute kidney injury: Glomerular filtration is a result of glomerular blood flow. The size of the kidney filtration fraction is determined by the effective arterial blood volume and the regulation of the vascular resistance of the afferent and efferent arterioles. The relaxation of the glomerular afferent arterioles and the contraction of the efferent arterioles provide for the maintaining of, and the autoregulation of, intraglomerular pressure and preserving GFR and urine output. The relaxation of afferent arterioles is controlled by the prostaglandins, while the contraction of the efferent ones is regulated by angiotensin II [36,37]. Thus, drugs exerting antiprostaglandin activity (NSAIDs) or affecting the renin–angiotensin–aldosterone system (angiotensin-converting enzyme inhibitors—ACEIs, angiotensin receptor blockers; sartans—ARBs) may significantly interfere with the aforementioned regulatory mechanism. Therefore, the use of those drugs may be associated with abolishing the correct mechanisms of glomerular filtration and an increased risk of acute kidney injury (AKI) development. In the case of NSAIDs, the abolishing of the vasoactive effect of prostaglandins is another complementary mechanism connected with the complex nephrotoxic effect of this class of drugs, as mentioned in the previous paragraph.

Taking into account drugs interfering with the RAA system, it should be emphasized that both ACEI and ARB are seldom truly nephrotoxic, although their effect on intrarenal hemodynamics can cause a reversible reduction in the glomerular filtration rate (GFR), without structural damage, and eventually, ultimately AKI development. Such a damaging effect on the kidneys is potentially possible in patients with an already existing impairment of glomerular autoregulation, based on the prostaglandin-mediated vasodilation of the afferent arteriole and angiotensin-mediated vasoconstriction of the efferent arteriole; congestive cardiac failure, chronic kidney disease, or in the setting of reduced renal perfusion (e.g., bilateral renal artery stenosis: severe hypotension; severe intrarenal arteriosclerosis) [38]. On the other hand, however, both ACEIs or ARBs are also nephroprotective and administered in patients with kidney damage in the course of diabetes, congestive heart failure, or hypertension to reduce the pathological remodeling of the kidney vessels. Moreover, there is still no clear and comprehensive assessment of the actual clinical relevance of the role of ACEI/ARB in the pathogenesis of AKI and recurrent AKI. Patients diagnosed with AKI are not treated further with ACEI/ARB to avoid the recurrence of this disorder. That said, the available clinical data are ambiguous and do not fully justify such a procedure [39]. Therefore, further studies are required to establish the role of ACEI/ARB in nephroprotective treatment following an episode of AKI.

The nephrotoxicity potential of ACEIs/ARBs may also result from drug interactions. The simultaneous chronic use of ACEI/ARB and NSAID is an example of possible drug interaction with a high potential for nephrotoxicity, especially in elderly patients. The risk is even increased when a diuretic agent is also administered (“triple whammy interaction”). One observational study analyzed the frequency of adverse reactions in patients over 75 years of age with multiple diseases receiving polypharmacotherapy. In the study, 12 patients were prescribed the ACEI/NSAID combination. It was then revealed that two of them developed acute renal failure, of whom one died and the other recovered after the discontinuation of both drugs. Moreover, four patients showed deterioration in renal function, which returned to normal after at least one of the drugs was stopped. Renal function remained stable in six patients. Therefore, it can be concluded that in elderly patients, ACEI and NSAID should not be used chronically, especially with an additional combination of diuretics [40]. In another literature review, the interactions between NSAIDs and diuretics, with or without additional renin-angiotensin aldosterone agents, were found to have the strongest association with the development of AKI [41].

Moreover, some drugs (calcineurin inhibitors, e.g., cyclosporine A) may lead to the vasoconstriction of the afferent vessels in a prostaglandin-independent manner, also leading to kidney function impairment [10,17,18,19,20,21]. The detailed description of the pathogenesis of cyclosporin A-induced nephrotoxicity involves both an acute, hemodynamically mediated, reversible phase and chronic renal functional deterioration as a result of irreversible and progressive tubulointerstitial injury and glomerulosclerosis. Acute vascular dysfunction results from an increase in vasoconstrictor factors (endothelin, thromboxane, the renin-angiotensin system), as well as a reduction of vasodilator factors (prostacyclin and nitric oxide). Moreover, ROS formation and sympathetic nerve activation in the kidneys, which increases renal vascular resistance, also play a role in the acute phase of cyclosporin-related nephrotoxicity. Chronic cyclosporin A nephropathy is associated with pathological kidney remodeling, irreversibly involving all three compartments of the kidneys: vessels (arteriolar hyalinosis), tubulointerstitium (tubular atrophy and interstitial fibrosis), and glomeruli (thickening and fibrosis of Bowman’s capsule and focal segmental or global glomerular sclerosis). A combination of cyclosporine-induced hemodynamic changes and the direct toxic effects of cyclosporine on tubular epithelial cells is thought to play a pathophysiological role in chronic phase development [42,43].

Tubular cell toxicity and acute tubular necrosis: As mentioned in the introduction, the proximal tubules are particularly vulnerable to the effects of nephrotoxins, including drugs. Drug-related tubular injury is triggered after drug reabsorption and their entering the tubular cells. Intracellularly, drugs may cause mitochondrial dysfunction, which impairs the energy management in the tubular cells, disrupting their integrity and adherence to the basement membrane. There is also the over-expression of adhesive proteins and increased oxidative stress. The increase in intracellular calcium concentration activates the proteases, which initiates the pathway involved in inflammation development and cell death through apoptosis or necrosis. Moreover, the transport functions of cells are disturbed, and the filtrate leaks back from the tubular lumen into the interstitial tissue. Ultimately, the tubular cells are exfoliated into the lumen of the tubules and form obstructive conglomerates, which increase intra-tubular pressure and inhibit GFR. Finally, a complex set of pathophysiological disorders known as acute tubular necrosis (ATN) develops. Drugs that are considered to exert their complex nephrotoxic action in such a way include aminoglycosides, amphotericin B, antiretroviral drugs, cis-platinum, iodinated contrast agents, foscarnet, and bisphosphonates [10,17,18,19,20,21].

It is estimated that up to 25% of all patients treated with aminoglycoside develop nephrotoxicity. Aminoglycosides exert their noxious effects as renal tubular toxicity, reduced glomerular filtration, and a reduction in renal blood flow. Aminoglycosides are polycationic agents that are subject to apical tubular uptake from the urinary space via endocytosis/pinocytosis after binding to megalin/cubulin receptors. These drugs are then translocated into lysosomes (followed by the inhibition of degrading enzymes), the Golgi body, the endoplasmic reticulum, and inside the mitochondria where they induce apoptosis and necrosis. Lysosomal injury is associated with membrane and organelles damage (“myeloid bodies”), increased oxidative stress, and mitochondrial dysfunction. Finally, energy failure in the tubules develops and contributes to the loss of their integrity and disturbances of numerous transmembrane transporters impairing tubular reabsorption. In the glomerulus, aminoglycosides increase intracellular calcium levels and induce mesangial smooth muscle contraction, which leads to decreased GFR. The reduction in renal blood flow is caused by increased vascular resistance in the renal vascular bed, resulting from endothelin and thromboxane release [8,44,45].

Amphotericin B is an anti-fungal agent used in many systemic infections, and this drug has high nephrotoxic potential. The mechanism of kidney damage induced by amphotericin B involves directly affecting the cell membrane resulting in increased permeability, as well as indirect effects produced by the activation of intrarenal mechanisms (tubuloglomerular feedback), the release of mediators (thromboxane A2), and changes in intracellular calcium levels. The most known consequences of amphotericin B therapy are tubular damage and the acute decrease of renal blood flow and filtration rate leading to AKI [46,47].

Antiretroviral drugs are another example of tubulopathy-potential agents. Treatment with acyclovir, foscarnet, tenofovir, adefovir, cidofovir and didanosine, lamivudine, stavudine, or zidovudine have all been associated with a risk of ATN development in HIV-infected patients. On the other hand, the administration of protease inhibitors (e.g., indinavir, atazanavir, nelfinavir, saquinavir, lopinavir, or ritonavir) is associated with the risk of intratubular precipitation of drug metabolites, due to their poor solubility [48,49].

Tenofovir, as a nucleotide reverse transcriptase inhibitor, is a substrate for the organic anion transporter 1 (OAT-1) located in the proximal tubular basolateral membrane. This drug accumulates in the proximal tubular cells and causes mitochondrial toxicity, likely through the depletion of mitochondrial DNA, leading to energetic failure, cellular dysfunction, and/or death. Consequently, proximal tubule damage is the most recognized form of the nephrotoxicity of antiretroviral drugs, and this dysfunction usually improves after tenofovir withdrawal. However, only 40% of patients achieve complete renal recovery [50].

Cisplatin enters the renal tubules after uptake by basolateral transporters (mostly proximal tubule organic cation transporter 2 -OCT2), and intracellularly, it is able to activate signaling pathways leading to cell apoptosis (MAPK, p53). Moreover, cisplatin causes increased oxidative stress and induces TNF-α production in the tubular cells, which triggers a robust activation of intrinsic and extrinsic apoptotic cascades and endonucleases. Many of these same pathways contribute to the cytotoxic actions of cisplatin in tumor cells. Cisplatin may also induce vasocontraction in the renal vasculature, leading to ischemic tubular cell death and a decreased glomerular filtration rate (GFR). Taking all of the above issues together, these pathological events may result in AKI development [51,52].

Iodine contrast agents are significant nephrotoxic compounds. Estimates indicate that they account for up to 11% of AKI episodes in hospitalized patients, especially those with pre-existing renal dysfunction or diabetes mellitus, in whom a standard hydration protocol was not administered during imaging [53,54]. Despite the extensive use of contrast agents in diagnostic procedures, the detailed pathogenesis of their nephrotoxic effects is not yet fully understood, and a combination of renal ischemia and direct toxic effects on the renal tubular cells are the main recognized mechanisms. The changes in renal hemodynamics induced by contrast media are due to the increased action of renal vasoconstrictors (vasopressin, angiotensin II, dopamine-1, endothelin, and adenosine) and the decreased activity of renal vasodilators (nitric oxide and prostaglandins). The consequence of these disturbances to kidney perfusion is hypoxic injury, and the most vulnerable kidney region is the deeper portion of the outer medulla, an area remote from the vasa recta supplying the renal medulla with blood, as this region is characterized by relatively high oxygen requirements due to salt reabsorption. Moreover, additional factors that may contribute to decreased renal blood flow include rheologic alternation–increased viscosity of contrast media and increased erythrocyte aggregation, with subsequent diminished oxygen delivery. The direct toxic effects on the renal tubules exerted by contrast agents are due to a reduced antioxidant enzyme capacity in the kidney and the direct cytotoxic effects mediated by the overproduction of oxygen free radicals. Additionally, the nonspecific effects of hyperosmolality should be mentioned because the administration of contrast agents may evoke osmolar-driven solute diuresis with the activation of tubuloglomerular feedback or an increase in tubular hydrostatic pressure, which may cause a compression of the intrarenal microcirculation and a decreased GFR [54,55].

Bisphosphonates are regarded to be the class of drugs with an attributed low incidence of adverse kidney effects. After dosage, between 27% and 62% of these drugs bind to bone minerals, and the remainder is not metabolized, being excreted via the kidneys; predominantly by both passive glomerular filtration and active transport in renal proximal tubular cells. Although bisphosphonates are generally well tolerated, the demonstration of their kidney adverse events, revealed in postmarketing clinical studies, has resulted in the inclusion of “warnings” on the prescribing information of all bisphosphonates, regarding the use of these agents in patients with severe renal impairment (CrCl <35–30 mL/min). The nephrotoxicity of bisphosphonates (predominantly induced by zoledronate or pamidronate) is mainly dependent on the route of administration; given orally, bisphosphonates are not associated with significant nephrotoxicity, however, nephrotoxicity is a potential limiting factor to the use of intravenous (IV) bisphosphonates, and their nephrotoxicity is both dose-dependent and infusion time-dependent. Pamidronate use has been associated with nephropathy based on focal segmental glomerulosclerosis, whereas zoledronate has mostly been associated with direct tubular toxic effects [56,57,58].

Crystal nephropathy: Kidney damage may also result from the precipitation and deposition of insoluble compounds, forming crystals and fine concretions within the tubular fluid, after exceeding their solubility; this, however, depends on the urinary concentration of the poorly soluble drug and its metabolites, as well as the urinary pH. This phenomenon occurs mainly in the distal tubules and initiates secondary changes in the renal interstitium. Drugs regarded as being associated with an increased risk of the ability to form intrarenal crystal deposition mostly include sulphonamides, triamterene, antiretroviral drugs used in HAART therapy (delavirdine, efavirenz, nevirapine, and rilpivirine), antacids (magnesium trisilicate, aluminum hydroxide), methotrexate, quinolones (ciprofloxacin, norfloxacin), and some antibiotics (e.g., ampicillin, amoxicillin) [10,17,18,19,20,21]. Detailed information on the etiopathogenesis of drug-related nephrolithiasis, with particular emphasis on the specific types of drug-induced stones, is beyond the scope of this study and can be found in the detailed reviews on this subject [59,60,61]. It should be emphasized that apart from drug-induced kidney stones, developing as a result of the direct precipitation of drugs and their metabolites in the urinary tract, other indirect “metabolic, drug-related stones” may be distinguished. The pathogenesis of the latter is associated with the induction of metabolic changes promoting lithogenesis caused by some drugs, and maybe a consequence of loop diuretics or laxatives that cause electrolyte changes, high doses of vitamin C that significantly acidify the urine, or xanthine oxidase inhibitors (allopurinol), and uricosuric compounds that cause hyperuricemia [60]. From a clinical perspective, crystal nephropathies are associated with abnormal urinalysis and urinary sediment findings, tubulopathies, and chronic kidney disease. Careful examination of urine sediment is often helpful in evaluating and preventing the possibility of developing crystal-related kidney injuries [62]. The massive accumulation of stones in the urinary tract can also lead to an increase in intra-tubular pressure that exceeds the glomerular pressure and results in a significant reduction of GFR. The final consequence of this may be obstructive AKI and kidney failure [63].

Rhabdomyolysis: Rhabdomyolysis is defined as a pathological condition of skeletal muscle cell damage, leading to the release of toxic intracellular material into blood circulation. The key pathophysiological finding in this disorder is an increase of intracellular free ionized calcium due to either cellular energy depletion or direct plasma membrane rupture. Initially, the increased calcium level contributes to the intensification of the skeletal muscle cell contractility. Finally, the consequence of the increased intracellular calcium is the activation of several proteases, mitochondrial dysfunction, and increases in the production of reactive oxygen species, ultimately resulting in skeletal muscle cell death and release of their content into the blood [64]. Clinically, rhabdomyolysis presents a triad of symptoms: severe muscular pain (myalgia), weakness, and myoglobinuria, manifested as the classically described tea-colored urine. However, the full triad of symptoms is observed only in <10% of patients, and >50% of patients do not complain of muscle pain or weakness, with the initial symptom being discolored urine. An elevated creatine kinase (isoenzyme CK-MM) level is the most sensitive laboratory indicator for evaluating any potential muscle injury leading to rhabdomyolysis (provided that concomitant heart or brain diseases are excluded) [65]. Massive skeletal muscle injury causes the release of a large amount of myoglobin that undergoes glomerular filtration due to the fact that the excess amount of myoglobin is not bound by the plasma haptoglobin and remains a free fraction. Rhabdomyolysis develops as a consequence of severe trauma, prolonged muscle ischemia, metabolic disorders (diabetic ketoacidosis, hypothyroidism, hyperaldosteronism), electrolyte disturbances (hypocalcemia, hypophosphatemia), intense physical activity, prolonged convulsions, alcohol or psychoactive agents abuse, and some noxious chemical or biological factors (e.g., animal venoms, arsenic, mercury, and some drugs). Myoglobin and other low molecular weight proteins precipitate in the tubular lumen causing the obstructive disturbances mentioned above. Moreover, myoglobin exerts direct nephrotoxic effects because the protein is reabsorbed by the tubules and acts inside them as a strong inductor of oxidative stress, cell death, and the release of pro-inflammatory mediators. Myoglobin also contributes to the low bioavailability of nitric oxide, with the subsequent deregulation of factors involved in the control of the vascular tone, such as endothelin-1, thromboxane A2, tumor necrosis factor, and isoprostanes [64,66]. The main recognizable drugs regarded to be causative factors of rhabdomyolysis are stains, however, there are more agents that are implicated in drug-induced rhabdomyolysis [10,17,18,19,20,21].

To sum up, any drug which directly impairs the production or use of adenosine triphosphate (ATP) in the skeletal muscle, or increases their energy requirements, which cannot be compensated for due to inefficient production, or alters calcium metabolism by the sarcoplasmic reticulum, may be an etiological factor of rhabdomyolysis. In addition, drug-induced indirect mechanisms of rhabdomyolysis may be distinguished, including ischemia, as a result of prolonged immobilization from drug overdosage with CNS depressants, thus impairing the delivery of oxygen and nutrients, drug-induced delirium, choreoathetosis, or dystonic reactions and seizures (which increase muscle activity and the demand for ATP) [67]. Thus, drugs that evoke central nervous system depression can cause prolonged immobilization, muscle compression, and tissue ischemia that results in myocyte injury; for example, narcotics, benzodiazepines, cyclic antidepressants, antihistamines, ethanol, glutethimide, and barbiturates may predispose the user to the development of rhabdomyolysis. In addition, drugs capable of causing neuroleptic malignant syndrome, characterized by the gradual development of hyperthermia, muscle rigidity, autonomic instability, an altered mental state, myoglobin, and elevated serum CK may be causative factors of drug-induced rhabdomyolysis. Drugs that cause neuroleptic malignant syndrome include phenothiazines, butyrophenones, antipsychotics, narcotics, and antidepressants. There are also various drugs that induce rhabdomyolysis through other mechanisms. Hypokalemia caused by diuretics, mineralocorticoids, or amphotericin B can predispose a patient to rhabdomyolysis. Corticosteroids appear to have a direct toxic effect on skeletal muscle, as seen in severe asthmatics who develop rhabdomyolysis. Overall, estimates indicate that approximately as many as 150 drugs may be associated with an increased risk of developing rhabdomyolysis [68].

Thrombotic microangiopathy: Thrombotic microangiopathy (TMA) is a clinical entity resulting in excessive and uncontrolled thrombosis in capillaries and arterioles induced by endothelial injury. TMA encompasses a heterogenous group of disorders characterized by microangiopathic hemolytic anemia, thrombocytopenia, and microthrombi leading to occlusive vascular thrombosis and ischemic damage or even infarction of the end organ (heart, brain, kidneys). The primary forms of TMA are thrombotic thrombocytopenic purpura (TTP) and hemolytic uremic syndrome (HUS) [69,70]. Drug-induced TMA follows the general mechanism of TTP and results from platelet activation and their degradation. Two main mechanisms can be distinguished as being immune and non-immune. The first of these is based on the identification of drug-dependent antibodies to platelets or other cells. These antibodies may react with platelets and form intraluminal microvascular aggregates. Moreover, neutrophils or endothelial cells may also form antigen-antibody complexes. Some drugs may exert direct endothelial activity and lead to increased platelet aggregation, with the overactivity of complementary systems and the release of clotting factors. The kidney vascular bed may also be affected during TMA. Among drugs attributed with the ability to induce thrombotic microangiopathy, antiplatelet agents (clopidogrel, ticlopidine), cyclosporine, mitomycin C, and quinine are mostly mentioned [10,17,18,19,20,21]. Moreover, other drugs capable of inducing TMA include some anti-infective agents (trimethoprim, sulfamethoxazole, fluoroquinolones, metronidazole, vancomycin), calcineurin inhibitors (cyclosporin A, tacrolimus), and other immunosuppressants (sirolimus, interferon α/β), monoclonal antibodies (muromonab-CD3, emicizumab, adalimumab, golimumab, certolizumab pegol, and VEGF inhibitors–bevacizumab, sunitinib), and drugs used in cancer treatment (gemcitabine, oxaliplatin, mitomycin, proteasome inhibitors–bortezomib, carfilzomib, ixazomib). TMA episodes were also reported in patients treated with valproic acid or in cocaine or opioid addicts, as well as in patients receiving intravenous immunoglobulins [70,71,72,73]. Antibody-mediated TMA has been confirmed for quinine, oxaliplatin, and vancomycin. On the other hand, the dose-dependent and cumulative toxicity model was adopted for cases of TMA connected to bevacizumab, levofloxacin, alemtuzumab, and interferon [73].

Table 2 presents and summarizes the drugs with marked nephrotoxicity potential.

## 4. The Molecular Basis of Drug-Induced Nephrotoxicity and the Role of Cytokines in the Development of this Disorder

For many drugs, the molecular mechanism of their nephrotoxic action has been clarified. The common, complex, interconnected elements of nephrotoxic action at the molecular level involve the activation of signal transducers and intracellular messengers, DNA damage, mitochondrial dysfunction, oxidative stress, sustained inflammatory response, and the activation of apoptotic pathways. Pathomechanisms of nephrotoxicity at the molecular level can be traced to the example of cisplatin. It seems that cisplatin-induced kidney damage has been described in the largest and most detailed way.

Cisplatin is predominantly excreted by the kidneys and the drug accumulates in the kidneys. This drug affects mostly proximal tubules, and glomeruli and distal tubules are affected subsequently. The concentration of the drug in tubular epithelial cells is five times greater than in blood. Therefore, even non-toxic blood levels may reach toxic levels in the kidneys. After filtrating into the urine, cisplatin enters the tubular cells via passive diffusion or transport-mediated facilitated diffusion with the participation of basolateral organic cation transporters (OCT), and reaches high concentrations in the proximal tubular cells of the inner renal cortex and outer medulla (S3 segment). Inside the tubular cells, cisplatin forms cross-links within and between DNA chains leading to the activation of various cellular responses, including the signaling of DNA damage, cell cycle checkpoints, DNA repair, and cell death. The drug also accumulates in the mitochondria of tubular cells and impairs mitochondrial bioenergetics due to the inducement of oxidative stress, and the release of pro-apoptotic factors, which ultimately lead to renal tubular cell death [52,74,75]. A detailed description of the basis of oxidative stress is beyond this review and it can be found in some papers focusing on this topic [76,77,78,79], including one of my previously published reviews [80]. However, to sum up, it should be concluded that reactive oxidative and nitrosative species contribute to the damage to kidney cell structure and their function, including lipid peroxidation, protein nitration and oxidation, enzyme inactivation, and DNA breaks. Finally, the signaling for the activation of apoptotic pathways is upregulated, causing kidney damage and cell death. Cisplatin also affects mitochondrial respiratory complexes and their function. The drug-induced mitochondrial dysfunction results in decreases in membrane electrochemical potential, substantial reductions in mitochondrial calcium uptake, and the depletion of mitochondrial antioxidant defense systems. Moreover, inflammatory mechanisms are also strongly linked to drug-induced nephrotoxicity due to the fact that oxidative stress and inflammatory processes are interrelated-pro-inflammatory mediators initiate oxidative stress and, on the other hand, free radicals are treated as one of the mediators initiating and maintaining inflammation. Cytokines play an important role in the molecular pathogenesis of drug-induced nephrotoxicity, especially TNF-α, which orchestrates the secondary release of interleukin-1, 4, 6 (IL-1β, IL-4, IL-6), transforming growth factor-β (TGF-β) and monocyte chemotactic protein-1 (MCP-1) [74,75]. The consequence of the chronic inflammation of the kidneys is also the process of renal tissue fibrosis, with particular importance in this process of TGF-β. It is a cytokine mainly contributing to the activation of renal tissue fibrosis processes by upregulating matrix protein synthesis, inhibiting matrix degradation, and altering cell-cell interaction [81,82]. Additionally, TNF-α induces the expression of adhesion molecules, including intercellular adhesion molecule 1 (ICAM-1), vascular cell adhesion molecule 1 (VCAM-1), and E-selectin, promoting an inflow of inflammatory cells in kidney tissues. The overproduction of TNF-α as a “key player” in the downstream regulation of the inflammatory response is related to the nuclear factor kappa B (NF-κB) pathway. Nephrotoxic agents, including cisplatin, activate phosphorylation and the subsequent translocation of NF-κB to the nucleus, finally leading to the increased transcription of specific genes encoding inflammatory mediators, including TNF-α. Thus, the increased expression of the cytokine in kidney tubular cells triggers their damage and death directly through TNF receptor type 1 (TNFR1) and indirectly by mounting a strong inflammatory response through TNF receptor type 2 (TNFR2) [83,84,85].

Similar molecular abnormalities to those seen with cisplatin nephrotoxicity have been reported for other drugs, e.g., aminoglycosides (gentamycin, kanamycin, streptomycin, and tobramycin), amphotericin B, antiviral agents (adefovir, cidofovir, and tenofovir) or radiocontrasts because those agents are responsible for AKI development, in the form of acute tubular necrosis, with oxidative stress and necroinflammation as the main underlying mechanisms [86].

In conclusion, taking into account the molecular description of drug-induced kidney damage, it is important to emphasize the importance of the inflammatory process and the increased oxidative stress ongoing in the kidney tissues, with the release of mediators (especially oxygen radicals, TNF-α, TGF-β) sustaining the course of inflammatory reactions and as a consequence of pathological damage and tissue remodeling.

## 5. Preventive Factors of Drug-Induced Nephrotoxicity

The most important and obvious preventive factor in reducing the incidence of drug-induced kidney dysfunction is the knowledge of the nephrotoxic potential of the drugs used in the treatment of the patient and the ability to predict the consequences of pharmacotherapy on kidney function. The early and proper recognition of nephrotoxicity symptoms is of key importance and allows for rapid therapeutic intervention and even the withdrawal of the drug used so far. Drug-induced nephrotoxicity, similarly to other kidney diseases, can be diagnosed with simple blood and/or urinary tests, including blood creatinine and urea nitrogen measurement; the estimation of GFR by creatinine clearance; and qualitative and quantitative evaluation of the blood/urine for new biomarkers indicating kidney function. So far, the greatest experience in experimental research, in a clinical setting, is associated with selected protein markers such as cystatin C (a marker of GFR), nephrin, podocin (podocyte proteins; markers of glomerular filter membrane integrity), kidney injury molecule-1 (KIM-1), neutrophil gelatinase-associated lipocalin-1 (NGAL-1), liver type fatty acid binding protein (FABP), and osteopontin (parameters reflecting tubulointerstitial damage). These laboratory parameters are increasingly used in the diagnosis of either AKI or CKD. Moreover, certain markers such as asymmetric dimethylarginine, inflammatory/fibrosis parameters (e.g., monocyte chemoattractant protein, transforming growth factor-b1), and Klotho-FGF23 axis raise the most interest as the best selective markers of CKD. A detailed description of the diagnostic application of these markers is beyond the scope of this paper and can be found in numerous reviews dealing specifically with this issue [87,88,89,90,91]. Due to simple laboratory assessment of kidney function, based on blood urea nitrogen (BUN) and creatinine measurement, being an imperfect marker of kidney function, as they are influenced by many renal and non-renal factors, some of the above-mentioned proteins have been proposed as more selective laboratory parameters of kidney dysfunction. Urine albumin and urinary kidney injury molecule-1 (KIM-1), β2-microglobulin (B2M), cystatin C, clusterin, and trefoil factor-3 (TFF-3) have all been accepted by the Food and Drug Administration (FDA) and European Medicines Agency (EMA) as specific biomarkers to monitor drug-induced nephrotoxicity, both in preclinical studies and on a case-by-case basis in clinical trials. The list of currently used laboratory markers believed to specify the presence of drug-induced kidney damage is presented in Table 3.

In clinical assessment, drug-related nephrotoxicity can manifest with AKI (in prerenal, intrinsic, and obstructive forms), nephrotic syndrome, renal tubular dysfunction, or CKD symptoms. Thus, it is necessary to carefully assess the clinical symptoms appearing in the patient because many kidney disturbances in the initial phases are mildly symptomatic and may be overlooked. General principles for reducing drug-induced nephrotoxicity involve identifying patients at increased cumulative risk of nephrotoxicity and then continuously monitoring kidney function in treated patients, especially when introducing any drug to a patient that is known to be nephrotoxic [95,96].

The most important issue of safe pharmacotherapy is the individualization of drug dosage, reducing the risk of adverse drug reactions, including kidney damage. Hence, it is crucial to precisely determine the doses of drugs used in the patient, adjusting them to kidney and liver functions. This is possible based on the determination of the individual GFR values in each patient undergoing pharmacotherapy. It is also worth mentioning that most drugs eliminated in the urine do not require dosage adjustment until the creatinine clearance declines below 50 mL/min [10]. The baseline kidney function in terms of GFR can be estimated with basic endogenous markers, e.g., creatinine, cystatin C, or β-2 microglobulin. There are some clinical conditions that cause their variations in patients, such as their muscle mass (creatinine) or fat mass, whether they smoke, any thyroid and corticoid disorders (cystatin C), lymphoproliferative and plasma cell disorders, and inflammation (β-2 microglobulin) [97]. GFR is routinely estimated with many formulas, for example, the Cockcroft–Gault formula, the Modification of Diet in Renal Disease (MDRD) formula, the pediatric Schwartz formula, or the Chronic Kidney Disease Epidemiology Collaboration (CKD-EPI) formula [10,97]. All the above-mentioned formulas for estimating GFR are based on the concentration of creatinine in the blood, whereas it is further influenced by muscle mass and creatinine metabolism. Thus, all equations to estimate GFR from serum creatinine include surrogates for muscle mass, such as age, sex, race, height, or weight [98]. Therefore, the search for new, more precise markers of glomerular filtration is still ongoing. Currently, it is also proposed to more comprehensively assess GFR value based not only on the blood creatinine levels. As mentioned above, there are efforts to facilitate the increased and routine use of cystatin C, especially to confirm estimated GFR in patients who are at risk of or have CKD, because combining filtration markers (creatinine and cystatin C) is more accurate and would support better clinical decisions than either marker alone [99]. Therefore, in diminishing drug-related kidney dysfunctions, it is crucial to adjust drug dosages to the current GFR value and, if possible, to avoid drugs with high nephrotoxic potential, as well as nephrotoxic drug combinations, leading to the interactions that increase the risk of nephrotoxicity; and to correct potentially modifiable risk factors, and preferentially use drugs which are non-nephrotoxic [9,21]. Ensuring the proper hydration of the patient is also important to maintain adequate kidney perfusion. Moreover, the current volume status of the patient should be assessed, monitored, and corrected, if need be, before the introduction of the nephrotoxic drug, in order to facilitate its elimination. There is no clinical consensus on how to provide the optimal solution, volume, or timing of fluids to restore and optimally sustain kidney perfusion to ensure the maintenance of an appropriate rate of drug excretion, which indirectly preserves its specific, safe concentration in the blood. However, the symptoms of significant intravascular volume (and total body water) depletion, such as orthostatic hypotension, decreased skin turgor, fast pulse, infrequent and low volume urination, dry tongue, or poor capillary refill, suggest a disturbance of the body’s water balance and the need for compensation [100].

If it is necessary to use drugs with significant potential nephrotoxicity, detailed recommendations are developed to prevent their harmful effects on the kidneys. For example, reference can be made to the prophylactic use of antioxidant compounds (vitamin E, vitamin C, flavonoids, melatonin, N-acetylcysteine, luteolin, lycopene, or coenzyme Q10) that reduce free radical damage in the course of the use of drugs that increase oxidative stress in kidney tissues, e.g., during treatment with vancomycin [101], cyclophosphamide [102], colistin [103], cyclosporine A [104], tacrolimus [105], contrast media [106], and cisplatin [107]. Antioxidants, vitamins, and minerals were also demonstrated to prevent gentamicin-induced nephrotoxicity [108]. In an experimental study, strong antioxidant and nephroprotective activity were revealed for polyphenols (caffeic acid phenethyl ester, curcumin, quercetin, resveratrol, catechin, hesperidin, and ellagic acid [109]). There was also specific nephroprotective treatment when some nephrotoxic drugs were administered in an experimental study: cilastatin administration, a blocker of megalin receptors and inhibitor of dehydropeptidase-1, reduced nephrotoxicity associated with vancomycin and aminoglycosides. Similarly, probenecid, an inhibitor of kidney OAT transporters, reduced kidney damage induced by tenofovir or methotrexate. Cimetidine and magnesium block kidney OCT transporters, thereby reducing cisplatin-related nephrotoxicity. Vancomycin-induced kidney injury was reduced with concomitant treatment with fosfomycin because the agent is an inhibitor of lysosomal enzymes and decreases the intratubular transport of vancomycin [110]. Finally, a valuable diagnostic tool should be mentioned–therapeutic drug monitoring (TDM) in the blood, enabling precise control when maintaining drug levels in the optimal therapeutic range. At the same time, TDM enables precise dosage adjustments if necessary, which also significantly reduces the risk of nephrotoxicity. TDM significantly decreases adverse kidney effects exerted by antimicrobial, anticancer, anti-epileptic, or immunosuppressant therapies, as well as nephrotoxicity induced by drugs used as psychiatric entities [111,112,113,114].

## 6. Conclusions

Pharmacotherapy implemented for the patient must be effective, but also safe. Kidney damage may be an unintended but possible side-effect of treatment due to this organ being preferentially vulnerable to damage due to exposure to drugs and metabolites during their excretion stage. Hence, the development of drug-induced kidney damage is a possible complication, especially in patients with pre-existing kidney dysfunction and other risk factors. The risk of drug-related nephrotoxicity significantly increases in patients with either primary kidney diseases or numerous diseases outside the kidneys and undergoing polypharmacy with numerous interacting drugs. Each treatment must be individualized, with precise dosage selection based on the functional status of the patient’s kidneys and liver, and covered by the continuous monitoring of renal function, especially in the case of administering drugs with proven nephrotoxic effects, as discussed in this paper. It should be emphasized that some drugs may induce a nephrotoxic effect using several complementary mechanisms (e.g., NSAIDs may exert a nephrotoxic effect by influencing kidney perfusion, glomerulonephritis, or chronic tubulointerstitial inflammation development). Therefore, the use of many drugs, especially when administered chronically and in high doses, significantly increases the risk of kidney damage. Some substances, such as contrast agents (iodinated and gadolinium), lithium salts, cis-platinum, cyclosporin A, aminoglycosides, and amphotericin B, are characterized by a high intrinsic nephrotoxicity potential, and in the presence of such substances, particular vigilance and continuous monitoring of kidney function are required.

## Figures and Tables

**Table 1:** The main four phenotypes of drug-induced nephrotoxicity, based on their clinical presentation [11,12].

Phenotype	Clinical Characteristics	Criteria	
		Primary	Secondary
Acute kidney injury	Acute interstitial nephritis (AIN) Acute tubular necrosis (ATN)	Decline of serum creatinine by at least 50% from its peak level over 7 days after drug discontinuation or change in drug dosing within 2 weeks OR Rise in serum creatinine level that presents or progresses to AKI stage 2 according to KDIGO—2.0–2.9 times baseline that occurred within the previous 7 days	Oliguria: urine output ≤ 0.5 mL/kg/h for ≥ 12 h that indicates AKI stage 2 Non-oliguric presentation in pediatrics: > 500 mL/day; 1 mL/kg/hour for 24 h Urinalysis findings: granular and muddy casts suggesting ATN, urinary eosinophils, proteinuria Fractional excretion of sodium > 1% Negative ultrasound findings Clinical symptoms for AIN: fever, rash, joint pains
Glomerular disorder	Hematuria Proteinuria	Result of kidney biopsy confirming glomerular damage (within 4 weeks of drug discontinuation) AND Proteinuria, characterized by:at least 1 g of protein in a 24-h urine collection; urine protein to creatinine ratio > 0.8 urine albumin to creatinine ratio > 0.8 Urine protein test strip: 2+ (indicating 100–300 mg/dL of urine albumin) Hematuria, diagnosed by the presence of > 50 red blood cells in high-powered field	Clinical presentation of nephritic or nephrotic syndrome Culture-negative leukocyturia with the presence of > 50 white blood cells in high-powered field Erythrocyte or fatty urinary casts
Tubular dysfunction	Fanconi syndrome Phosphate wasting Renal tubular acidosis Diabetes insipidus SIADH	Hypophosphatemia OR glucosuria confirmed by urinalysis with 3+ glucose (without diabetes) OR Hyperchloremic metabolic acidosis with hypokalemia or hyperkalemia OR Diabetes insipidus: polyuria > 3 L/day; hypernatremia > 155 mEq/L on multiple occassions	Phosphaturia: fractional excretion of phosphates > 5%; urinary phosphates excretion > 100 mg/day Hypomagnesemia: serum magnesium level < 1.2 mg/dL Hypouricemia: serum uric acid level < 2 mg/dL
Nephrolithiasis	Crystalluria Ultrasound findings of kidney stones or gravel	The onset of symptoms of kidney stones or gravel follows drug exposure with no previous history of nephrolithiasis No evidence of congenital nephrolithiasis In obstructive nephrolithiasis: a rise in serum creatinine level that presents or progresses to AKI stage 2 according to KDIGO—2.0–2.9 times baseline that occurred within the previous 7 days In non-obstructive: urinalysis with crystals	Laboratory assessment of the composition of excreted urinary stones Analysis of electrolytes in urine

**Table 2:** The most commonly used nephrotoxic drugs that are etiological factors in drug-induced nephrotoxicity.

Clinical Entity of Nephrotoxicity	Drug Class	Drug Examples
Acute kidney injury	Non-steroidal anti-inflammatory drugs (NSAID)	Diclofenac, Naproxen, Ibuprofen, Indomethacin
	Other non-opioid analgetics	Acetaminophen, Aspirin
	Angiotensin-converting enzyme inhibitors (ACEI)	Benazepril, Enalapril, Fosinopril, Lisinopril
	Angiotensin II receptor AT1 antagonists (blockers; ARB)	Losartan, Valsartan
	Calcineurin inhibitors	Cyclosporine A, Tacrolimus
Acute tubular necrosis (tubular toxicity)	Non-opioid analgetics	Acetaminophen
	Anti-microbials	Aminoglycosides, Amphotericin B, Tetracycline
	Antiretrovirals	Adefovir, Cidofovir, Tenofovir
	Bisphosphonates	Pamidronate, Ibandronate, Alendronate
	Calcineurin inhibitors	Cyclosporine A, Tacrolimus
	Contrast dye	Gadolinium, Iohexol
	Miscellaneous	Acetazolamide, Cis-platin, Pentamidine
Glomerulonephritis	Miscellaneous	Gold compounds, Interferon-alpha, Hydralazine, Lithium, Penicillin G, Propylthiouracil
Acute interstitial nephritis	Biological drugs	Bevacizumab, Tyrosine kinase inhibitors (Sorafenib, Sunitanib)
	Anti-microbials	Beta-lactams, Cephalosporins, Fluoroquinolones, Rifampicin, Vancomycin, Sulfonamides
	Anti-epileptic drugs	Phenytoin, Phenobarbital, Carbamazepine
	Antivirals	Acyclovir, Indinavir
	Diuretics	Loops-Furosemide, Thiazides
	Histamine-2 antagonists	Ranitidine, Famotidine
	NSAID	Diclofenac, Naproxen, Ibuprofen, Indomethacin
	Proton pump inhibitors	Omeprazole, Pantoprazole, Lansoprazole
	Miscellaneous	Allopurinol
Chronic interstitial nephritis	Non-opioid analgetics	Acetaminophen, Aspirin
	NSAID	Diclofenac, Naproxen, Ibuprofen, Indomethacin
	Calcineurin inhibitors	Cyclosporine A, Tacrolimus
	Chinese herbs	Aristocholic acid
	Miscellaneous	Lithium
Crystal nephropathy	Anti-microbials	Ampicillin, Ciprofloxacin, Sulfonamides, Triamterene
	Antivirals	Acyclovir, Foscarnet, Ganciclovir, Indinavir
	Miscellaneous	Methotrexate
Rhabdomyolysis	Antidepressants	Amitriptyline, Imipramine, Doxepin
	Antihistamines	Diphenhydramine, Chlorphenamine, Promethazine
	Antipsychotics	Phenothiazines—Chlorpromazine, Fluphenazine, Butyrophenones—Haloperidol
	Corticoids	Dexamethasone, Triamcinolone
	Diuretics	Loops-Furosemide, Thiazides
	Inhalation anesthetics and muscle relaxants	Fluranes, Succinylcholine
	Psychoactive agents	Cocaine, Methamphetamine, Caffeine, Morphine
	Sedatives and anti-epileptics	Benzodiazepines, Barbiturates, Phenytoin
	Statins	Lovastatin, Simvastatin, Pravastatin
	Miscellaneous	Lithium, Sympathomimetics
Thrombotic microangiopathy	Antiplatelets	Clopidogrel, Ticlopidine
	Miscellaneous	Amitriptyline, Cyclosporine, Mitomycin C, Quinine

**Table 3:** The potential biomarkers of drug-induced kidney damage [87,92,93,94].

Localization of Nephron Segment	Proposed Biomarker
Glomerulus (filtration)	Beta-2-microglobulin Beta-trace globulin
Glomerulus (filtration barrier damage)	Podocin Nephrin Podocalyxin
Proximal tubules	N-acetyl-D-glucosaminidase Retinol binding protein Kidney injury protein-1 (KIM-1) Fatty acid binding protein (FABP) Neutrophil gelatinase-associated protein-1 (NGAL-1)
Distal tubules	Osteopontin Neutrophil gelatinase-associated protein-1 (NGAL-1) Clusterin
Tubulointerstitium	Interleukin-6 Interleukin-8 Interleukin-18 Monocyte chemoattractant protein-1 (MCP-1) Tissue inhibitor of metalloproteinases-1 (TIMP-1) Matrixmetalloproteinases 2 and 9 (MMP2, MMP9) Connective tissue growth factor (CTGF) Markers of oxidative stress: - Advanced oxidation protein products (AOPP) - Thiobarbituric acid reactive substances (TBARS) - Advanced glycation end products (AGE) - Malondialdehyde (MDA)

## Data Availability

Not applicable.
